# Digital pharmaka and the crisis of subjectivation: a longitudinal phenomenological study on the educational use of technology in higher education through the philosophy of Bernard Stiegler

**DOI:** 10.3389/fpsyg.2026.1989076

**Published:** 2026-09-30

**Authors:** Zafer Kadirhan

**Affiliations:** NeuroSimLab, Department of Software Engineering, Faculty of Engineering, Ankara University, Ankara, Türkiye

**Keywords:** digital technologies, higher education, hyperattention, pharmakon, subjectivation

## Abstract

This study investigates the ambivalent effects of digital technologies on the subjectivation processes of university students, drawing on Bernard Stiegler’s philosophy of technics and the concept of the pharmakon, technology as both remedy and poison. Using a longitudinal qualitative design grounded in interpretative phenomenological analysis (IPA), the research examines how digital technologies – including smartphones, social media, and AI tools – affect students’ attention, memory, desires, and sense of self over an academic semester. Data from three waves of in-depth interviews and reflective journals with twelve undergraduates reveal six interrelated themes: (1) from habitual engagement to cognitive embodiment: the internalization of digital technology; (2) the fragmentation of cognitive focus: the collapse of attention regimes; (3) loss of control and addiction-like engagement: perceived dependence on digital tools; (4) disconnection from meaning: digital acceleration and its impact on memory and meaning-making; (5) Erosion of subjective integrity: identity alienation in digital learning; and (6) reclaiming the Pharmakon: moments of remedial engagement. These findings demonstrate that digital technologies not only influence learning behaviors but also restructure the symbolic, affective, and libidinal foundations of student subjectivity, while also revealing fragile but meaningful counter-tendencies through which students reclaim symbolic production and collective desire. The study extends existing empirical research by integrating lived experience with Stiegler’s pharmacological lens, offering critical insights into the psychosocial and existential costs of algorithmic learning environments. It concludes by outlining implications for rethinking educational policy and pedagogy to restore attention, care, and symbolic richness in digitally mediated higher education.

## Introduction

Digital technologies have become infrastructural within higher education, reorganizing how students learn, communicate, and manage academic life. Smartphones, social media, learning management systems, and AI tools extend access to resources and make study more flexible, but they also cultivate habitual—sometimes compulsive—forms of engagement that are increasingly difficult to interrupt (Dontre, 2020; Chen et al., 2020; Kamalov et al., 2023; Aagaard, 2021). A growing body of research links these digitally saturated environments to depression, anxiety, academic burnout, cognitive overload, and persistent difficulties disengaging from platforms (Boursier et al., 2020; Mheidly et al., 2020; Twenge, 2020; Nan et al., 2024). These patterns frequently consolidate prereflectively, as ordinary routines, and only later become recognizable as problematic (Merleau-Ponty, 1962). Such prereflective consolidations are not merely individual tendencies but are shaped and stabilized by contemporary attention infrastructures (Stiegler, 2010; de la Torre et al., 2025). They therefore cannot be reduced to personal weakness or poor self-control; rather, they are partly shaped by the technical organization of everyday attention (Stiegler, 2010). In this study, “addiction-like” denotes a phenomenological pattern of dependency and loss of control that is structurally produced, keeping the focus on how environments organize attention, memory, and desire. From a Stieglerian perspective (Stiegler, 2013b), the issue is not primarily the quantity of use but the qualitative transformation of desire into drive. Engagement becomes repetitive, short-circuited, and detached from symbolic elaboration, learning, and collective meaning-making.

Bernard Stiegler’s philosophy of technics provides a framework for analyzing these dynamics. Technics is treated not as a neutral instrument but as a constitutive condition of human life—there is, in his terms, no human without technics and no technics without the human (Stiegler, 2010, 2013a, 2013b). Drawing on Derrida’s (1981) notion of the pharmakon—simultaneously remedy and poison—Stiegler develops a pharmacology of technics in which the same supports that enable learning and coordination can also become toxic when organized around industrial logics of capture. Through the concept of tertiary retention, he shows how technical systems of recording and inscription condition what is stored, resurfaced, and anticipated. These processes structure how subjects attend, remember, and imagine. Addiction-like engagement, in this sense, names a pharmacological configuration in which supports meant to enable individuation capture attention, exteriorize memory, and redirect desire toward repetitive, drive-based circuits rather than shared symbolic projects. Across his work on youth and education, Stiegler describes these developments as elements of a broader crisis of subjectivation, marked by symbolic misery—the erosion of shared cultural resources through which young people form desires of their own—and proletarianization, the loss of experiential know-how as knowledge and expertise are exteriorized into technical systems (Stiegler, 2010, 2013a, 2013b). In the context of this study, “subjectivation” does not refer to a static or pre-existing “student subjectivity,” but rather to the ongoing coming-into-being of subjectivity. Drawing on Stiegler (2010, 2013b), it is understood as a dynamic, relational process through which students continuously form their identities, critical capacities, and desires in interaction with their technical, social, and educational milieus. Understanding this process is crucial because digital infrastructures do not merely deliver information; they actively intervene in how this coming-into-being unfolds, often disrupting the symbolic supports necessary for independent thought. Higher education is thus understood not merely as an information system but as a privileged site of symbolic production and collective desire, where dialogue, critique, and shared study support processes of transindividuation. When digital infrastructures displace symbolic production with rapid symbolic consumption, these communal supports weaken, increasing vulnerability to repetitive, feedback-loop attachments.

Empirically, however, most research on problematic or addictive digital use in higher education remains large-scale and correlational, or focuses on individual traits and coping strategies. This literature has mapped important associations between screen time, psychosocial outcomes, and academic performance, and has begun to explore mediating mechanisms such as stress, resilience, and burnout (e.g., Hao et al., 2022; Yang et al., 2024). Yet it rarely connects students’ lived experiences of dependency and loss of control to the structural organization of attention, memory, and desire in digital infrastructures, or to the pharmacological ambivalence theorized by Stiegler.

The present study addresses this gap by bringing Stiegler’s primary works on technics, youth, and education into close dialogue with longitudinal, idiographic accounts of undergraduate students’ digital lives. Using longitudinal Interpretative Phenomenological Analysis (IPA), it traces students’ trajectories of dependency, loss of control, and attempts to regulate their use of smartphones, social media, and AI tools over the course of an academic semester. The analysis examines how digital infrastructures and institutional conditions organize addiction-like patterns of engagement, how these patterns are experienced and justified by students themselves, and what educational practices might support their reconfiguration toward care, symbolic production, and collective desire.

## Literature review

### Digital infrastructures and the reorganization of attention and memory

Over the past decade, accelerated by the COVID-19 pandemic, higher education has consolidated digital infrastructures as routine conditions of teaching, assessment, and academic coordination (Abbas et al., 2021; Daniela, 2021; Haleem et al., 2022). Smartphones, social media, AI applications, and learning management systems now support communication, collaboration, and flexible study, and are embedded in mainstream pedagogical models such as hybrid, online, and flipped classrooms (Bonfield et al., 2020; Turnbull et al., 2021; Skulmowski, 2023). Faculty increasingly rely on digital platforms for course design, content delivery, and evaluation, while students weave multiple tools into both academic and everyday routines (Hazaymeh et al., 2024; Nikolopoulou, 2022).

Treating these systems simply as neutral delivery channels obscures a deeper shift. Digital platforms operate as architectures of attention and memory. Interfaces, notifications, recommendation systems, and data-driven personalization not only transmit information but also format the temporal rhythms of study, task switching, interruption, and persistence. They can function as “attentional scaffolds” or “digital slot machines” that keep students engaged in continuous loops of checking and responding (Voinea et al., 2024). From a Stieglerian perspective, such systems can be viewed as configurations of tertiary retention—technical supports that record, store, and algorithmically process experience in ways that pre-select what is likely to be remembered and anticipated (Stiegler, 2010, 2013b). What appears to students as spontaneous curiosity or routine checking is already partly shaped by how platforms sort, rank, and resurface information. Digitalization, therefore, involves more than a change in teaching practices; it reconfigures the technical conditions under which learning and subjectivation occur.

### Psychosocial and cognitive consequences of digitally mediated academic life

Empirical work documents both benefits and harms of this reconfiguration. Digital tools can enhance access to resources, personalize learning pathways, and support motivation and self-regulation through feedback and varied materials (Ali et al., 2023; Asratie et al., 2023; Kasneci et al., 2023). Concurrently, digitally intensive environments have been associated with distraction, problematic use, and a persistent sense of connectivity, often conceptualized as fear of missing out (FoMO). This persistent connectivity has the potential to foster superficial learning and reduce opportunities for sustained reflection (Bedenlier et al., 2020; Lo Coco et al., 2020; Rozgonjuk et al., 2020). Psychologically, heavy engagement with platforms is associated with depression, anxiety, loneliness, and academic burnout among university students (Huang, 2020; Twenge, 2020; Yang et al., 2024).

From a pharmacological perspective, these outcomes can be read as symptoms of disrupted symbolic mediation. Stress, acceleration, and continuous solicitation erode the temporal supports required for reflection, learning, and collective meaning-making, particularly in data-driven ‘learning zones’ shaped by psychometric monitoring (Bates, 2021). Recent studies illuminate how these associations are configured. Hao et al. (2022), for example, show that academic burnout mediates the relationship between student stress and problematic smartphone use, with resilience acting as a moderating factor. Research on technostress similarly links digital demands to reduced academic productivity (Upadhyaya and Vrinda, 2021). Such findings suggest that addiction-like patterns of engagement emerge within broader constellations of stress, coping, and institutional demands rather than in a vacuum. Socially, digitally mediated interaction can both enable new forms of connection and weaken traditional forms of belonging and communal life within academic settings and beyond (González-Bailón and Lelkes, 2023; Kim and Yu, 2022; Mihelj and Jiménez-Martínez, 2020; Sum and Oancea, 2022; Smith D. et al., 2021).

At the cognitive level, multitool use has been linked to hyperattention, characterized by rapid task-switching, a preference for immediate stimulation, difficulties sustaining focus on a single task, with attention and distraction coexisting in persistent tension (Alho et al., 2022; Hamilton et al., 2023; Schmitt et al., 2021; Lee, 2021). These conditions also reflect increased extraneous cognitive load in digital learning environments (Skulmowski and Xu, 2021). Hyperattention has been theorized as an expression of the industrialization of attention in contemporary media environments (Bradley and Kennedy, 2019; Keller et al., 2020; Lee, 2019). Overall, evidence indicates that the psychosocial and cognitive effects of digitalization are unevenly distributed. Gender, socioeconomic status, and digital literacy shape both opportunities and vulnerabilities, amplifying existing inequalities and deepening digital divides (Ertl et al., 2020; Mathrani et al., 2021). This body of work robustly identifies correlates and some mechanisms of problematic digital engagement, but typically focuses on individual symptoms and outcomes. It rarely examines how digital infrastructures structurally organize attention, memory, and desire, thereby predisposing particular modes of attachment. It is precisely at this structural level that Stiegler’s pharmacology becomes analytically useful, framing addiction-like engagement as a technical failure of symbolic mediation rather than merely an individual deficit in self-control.

### Stiegler’s pharmacology of technics: from digital infrastructures to subjectivation

Stiegler’s philosophy of technics, developed in close dialogue with Derrida, offers a conceptual repertoire for articulating how digital infrastructures shape subjectivation. Building on Derrida’s (1981) reading of the pharmakon as both remedy and poison, Stiegler (2010, 2013b) argues that technics are constitutively pharmacological: the same supports that make human life and learning possible can also become toxic when captured by industrial logics that short-circuit care, critique, and collective meaning-making. For Stiegler, addiction is not an individual pathology but a technical failure of symbolic mediation. When tertiary retention is organized primarily around engagement metrics and continuous solicitation, desire is deprived of the temporal and collective supports required for learning, reflection, and individuation. Moore (2018) offers a pharmacological reading of addiction, analyzing how compulsive attachments emerge when technical supports meant to sustain care and reflection instead reinforce toxic patterns of repetition.

Within critical literature on digital media, education, and subjectivation, Stiegler’s work offers a systematic framework for analyzing how contemporary media infrastructures reorganize attention, memory, and symbolic life. In *Taking Care of Youth and the Generations* (Stiegler, 2010), he analyzes contemporary media as industrial organizations of attention that disrupt processes of transindividuation—the co-formation of subjectivity through shared symbolic practices and circuits of meaning. Extending this analysis, Bradley (2015), drawing directly on Taking Care of Youth and the Generations, conceptualizes contemporary media as systems of hyper-solicitation that continually capture and modulate youth attention and desire, reconfiguring the temporalities and rhythms of everyday life. By formatting experience through standardized cultural products and synchronized temporalities, these media environments risk producing symbolic misery, a thinning of symbolic resources and practices that undermines the formation of singular yet shared desires (Stiegler, 2010, 2013a). Within this context, youth are intensively solicited by media systems, while, at the symbolic level, they remain insufficiently equipped to translate these solicitations into durable and collective projects. In higher education, this concern appears in debates about how digital milieus reshape the conditions for critical thinking (Kerruish, 2025).

These concerns are further articulated in *What Makes Life Worth Living: On Pharmacology* (Stiegler, 2013b), where Stiegler develops the pharmacological logic underpinning this diagnosis. He argues that the curative potential of technics depends on their reinvestment in practices of care, education, and critique. When such reinvestment fails, technics contribute to a broader crisis of subjectivation, characterized by weakened symbolic mediation and diminished capacities for reflection and long-term orientation. Heaney (2020) extends these concerns to mental health, arguing that technics can erode the capacities of care and critique required to resist toxic configurations, particularly in educational and institutional contexts. Within this theoretical framework, proletarianization refers to the loss of experiential know-how and understanding as tasks, procedures, and memory are exteriorized into technical systems; individuals become operators of processes whose rationale they do not fully grasp (Stiegler, 2010, 2013b; Bates, 2021; Beime et al., 2023). Attention and memory are historically and technically mediated, not neutral inner capacities. Tertiary retention—the layer of technical recording and inscription that supplements individual and collective memory—plays a central role. In digital environments, tertiary retention takes the form of databases, logs, and algorithmic profiles that select, rank, and anticipate content for users. For students, what they see, revisit, and are reminded of is shaped by computational logics optimized for engagement rather than learning; platforms do not simply store past activity, they actively steer what students are likely to do next. Together, contemporary Stieglerian studies (e.g., Bradley, 2015; Moore, 2018; Heaney, 2020) provide the immediate theoretical ground on which the present study stands. They translate Stiegler’s primary concepts into accounts of digital media as pharmacological attention infrastructures that organize addictive attachments and vulnerabilities.

Higher education, from this perspective, is more than a system for delivering information. It is a key space where shared meanings and collective aspirations are formed. Through dialogue, critical discussion, and sustained study, learners participate in creating and reshaping meanings together. These symbolic and communal aspects of learning—such as communities of inquiry, interpretive work, and pedagogical relationships—make education central to the formation of subjectivity rather than a simple accumulation of knowledge (Stiegler, 2013a, 2013b). When digitalization shifts emphasis from symbolic production to rapid symbolic consumption, these communal supports weaken, increasing vulnerability to dependency and self-reinforcing patterns of engagement. The present study extends this work by focusing on longitudinal, idiographic accounts of undergraduate students’ digital lives; using longitudinal IPA, it traces trajectories of dependency, loss of control, and attempts to regulate use of smartphones, social media, and AI tools across an academic semester to examine how pharmacological dynamics are lived, narrated, and sometimes reconfigured in everyday academic life. This heuristic reliance on Stiegler is not intended as an uncritical application of a single framework; rather, it treats his concepts as productive starting points to be tested against, and where necessary complicated by, the empirical material itself.

## Method

### Research design

This study employed a qualitative, longitudinal design grounded in IPA to investigate how undergraduate students make sense of their engagement with educational technology over time. The longitudinal approach was integral to capturing both the stability and transformation of participants’ experiences, as it facilitated the examination of changes in meaning-making and patterns of technology use across multiple time points during the academic semester (Wood et al., 2023; Derrington, 2019). This design further allowed the study to trace trajectories of dependency, loss of control, and attempts at regulation as they unfolded in relation to smartphones, social media, and AI tools. IPA was selected for its strength in providing a detailed, idiographic analysis of lived experience within complex educational contexts, including prereflective and taken-for-granted aspects of everyday engagement and meaning-making (Smith et al., 2009, Smith J. A. et al., 2021). Within this longitudinal IPA design, key Stieglerian concepts—such as the pharmakon, tertiary retention, symbolic misery, proletarianization, and the crisis of subjectivation—were treated as sensitizing concepts that guided the interpretation in a heuristic rather than doctrinal manner (Stiegler, 2013a, 2013b). This heuristic stance reflects recent critiques of Stiegler’s framework (Bradley, 2015; Hayles, 2021), which caution against treating pharmacology as a totalizing explanation and call for greater attention to situated agency. This approach allowed for attention to both moments of resonance and points of tension in participants’ accounts. Methodologically, the analysis proceeded in two distinct stages: a participant-led experiential analysis, followed by a theoretically sensitized abductive interpretation (Timmermans and Tavory, 2012). Stiegler’s pharmacology of technics further informed the research, providing a critical theoretical lens for interpreting the ambivalent (pharmacological) impact of digital technologies on attention, memory, desire, and student subjectivity, as articulated in his analyses of youth, education, and digital pharmacology (Stiegler, 2010, 2013b). Within educational contexts, this perspective has been used to conceptualize how students are increasingly positioned as consumers of preformatted knowledge, with learning organized by technical systems rather than pedagogical relations (Forrest, 2019). Against this methodological and theoretical background, the study examines how higher-education students live and narrate addiction-like engagements with digital technologies when these are conceptualized as pharmacological configurations of attention, memory, and desire. Using longitudinal IPA, it traces trajectories of dependency, loss of control, and attempted regulation through the Stieglerian concepts outlined above, clarifying how digital infrastructures organize these patterns of engagement and how they may be reconfigured toward care, symbolic production, and collective desire.

### Participants

A purposive maximum variation sampling strategy was adopted to ensure diversity among undergraduate students. The study sample comprised twelve participants from different disciplines. Participants ranged in age from 19 to 24 years and were distributed across gender, residential background (urban or rural), socioeconomic status, and ethnic/national identities. All participants were active users of educational technologies, social media, and AI tools. Comprehensive information about the study was provided to each participant before data collection, and written informed consent was obtained. Confidentiality and the right to withdraw at any time were emphasized, and the study received ethical approval from the university’s institutional review board. All participant characteristics reported in Table 1 were self-reported and presented solely to enhance contextual understanding, rather than as variables informing the study’s analytical framework. Detailed demographic information for all participants is presented in Table 1.

**Table 1:** Demographic profile of participants (*n* = 12).

Participant	Gender	Age	Ethnic/National background	Socio-cultural background	Socio-economic class	Academic discipline	Technology usage level
P1	Female	20	National	Urban	Middle	Engineering	High
P2	Male	22	National	Rural	Working	Natural Sciences	Moderate
P3	Female	19	National	Urban	Upper-Middle	Education	High
P4	Male	23	International (Africa)	Urban	Middle	Health Sciences	High
P5	Female	21	National	Rural	Working	Engineering	Moderate
P6	Male	20	National	Urban	Middle	Natural Sciences	High
P7	Female	22	National	Rural	Working	Education	Low
P8	Male	21	National	Urban	Middle	Health Sciences	High
P9	Female	24	National	Urban	Middle	Engineering	Moderate
P10	Male	19	National	Rural	Working	Natural Sciences	Low
P11	Female	21	International (Europe)	Urban	Upper-Middle	Education	High
P12	Male	22	National	Rural	Middle	Health Sciences	Moderate

The term “National” denotes participants who hold citizenship of Türkiye. Technology Usage Level was self-reported on a three-point scale (low: <2 h daily; moderate: 2–5 h; high: >5 h) and is presented for descriptive context only, not as an analytical variable.

This diverse participant profile supported a contextualized interpretation of the participants’ lived experiences of technology dependence and alienation over the academic semester.

### Data collection procedure

Data were gathered through semi-structured, face-to-face individual interviews at the beginning (week 1), midpoint (week 8), and end (week 16) of the semester. Each session lasted 45–60 min, was held in a private setting conducive to open dialogue, and was audio-recorded with consent. Transcriptions were completed verbatim to ensure accuracy and preserve participants’ narratives. To enrich the dataset and enable methodological triangulation, participants submitted digital journals or written reflections throughout the semester. Eleven of the twelve participants contributed a total of 147 entries (*mean* = 12.30; *range* = 8–18). Optional prompts were provided (e.g., “Describe a moment this week when your use of technology felt supportive or difficult”), but participants were encouraged to write freely. Journals were analyzed alongside interview transcripts using the same idiographic procedure. The interview protocol was developed through an iterative literature review grounded in Stiegler’s theoretical perspective. In particular, prompts were designed to elicit experiences related to attention and distraction, memory and forgetting, loss of control, and meaning-making in learning, which align with Stiegler’s analyses of attention regimes, tertiary retention, proletarianization, and symbolic misery (Stiegler, 2010, 2013a, 2013b). Open-ended guiding questions prompted participants to elaborate on shifts in technology use, experiences of support and alienation, and perceived impacts of technology on learning and well-being. The protocol was pilot-tested with two students who were outside the main sample, leading to refinements that improved clarity and contextual sensitivity. All interviews were conducted in Turkish, the national language of Türkiye, the shared language of participants and the researcher. While some participants were non-native speakers of this language, all possessed sufficient proficiency for in-depth discussion. Analysis was conducted in this language to preserve metaphorical nuance and affective tone. Quotations were translated into English by the author and independently verified by a bilingual colleague through back-translation. The study was conducted at Ankara University, Ankara, Türkiye. The institution operates a Moodle-based LMS, and during the study period, no formal policy governed students’ use of generative AI tools. Course requirements regarding technology varied considerably across departments.

### Data analysis and theoretical sensitization

Data were analyzed using IPA, which facilitated a detailed exploration of how students made sense of their engagement with educational technology over time. Within this analytic approach, the analysis unfolded in two analytically distinct yet interrelated stages. It began with repeated readings of transcripts to ensure immersion in participants’ narratives. This iterative engagement reflects IPA’s epistemological commitment to meaning as co-constructed through the researcher’s sustained interpretive relationship with the data rather than extracted as fixed categories (Engward and Goldspink, 2020). Significant statements and affective nuances were annotated, and transcripts were segmented into meaning units and then descriptively coded. Codes were organized into themes and subthemes, enabling the identification of patterns across cases and time points. Consistent with IPA’s idiographic commitment, each participant’s three waves were first analyzed as an individual case; within-case themes were developed before any cross-case comparison. Only after completing this within-case procedure were convergence and divergence examined across participants (Smith J. A. et al., 2021).

In the first stage of analysis, themes were developed through close engagement with participants’ language and experience. While this stage remained grounded in participants’ accounts, the researcher’s familiarity with Stiegler’s work inevitably shaped the interpretive horizon; the analysis is therefore described as abductive rather than purely inductive (Timmermans and Tavory, 2012). At this stage, attention was directed to how participants described their everyday engagements with technology, including shifts in attention, emotion, and learning practices across the semester. The symbolic and communal dimensions of learning were treated not as optional interpretive layers but as core analytic criteria. Where students’ accounts revealed shared practices of dialogue, study, and collective orientation, these were read as instances of symbolic production and collective desire; where such practices were absent, fragmented, or explicitly mourned, this absence was analyzed as an empirical indicator of symbolic misery and crisis of subjectivation rather than as a mere lack of social interaction.

In the second stage, the emergent thematic structure was re-read in close dialogue with Stiegler’s primary texts, allowing his concepts to function as sensitizing analytic operators rather than abstract labels (Stiegler, 2010, 2013a, 2013b). Experiences of unstoppable scrolling, compulsive checking, and difficulty disengaging were interpreted through the pharmakon and tertiary retention as moments in which technical supports both enable and capture attention. Reports of meaninglessness, emotional numbness, or disconnection from study were examined through symbolic misery and crisis of subjectivation, while descriptions of “going through the motions” with limited understanding of why or how tasks were performed were analyzed in terms of the proletarianization of knowledge and know-how. Conversely, moments in which students reclaimed slower study practices, engaged in shared discussion, or pursued collaborative projects were interpreted as fragile reinvestments in symbolic production and collective desire. These mappings guided the IPA analysis of lived experience back to the structural organization of technics theorized by Stiegler. Where participants’ accounts did not fit neatly into these patterns—particularly where durable practices of care, study, or community were sustained within pervasive platform environments—such accounts were analyzed as counter-tendencies and conditions of possibility for pharmacological “remedy,” rather than treated as analytic noise. To avoid treating “*digital technologies*” as a monolithic category, the analysis distinguished three functional groupings based on participants’ accounts: attention-capturing technologies (smartphones, social media, short-form video), which were primarily associated with compulsive checking and attentional fragmentation; cognitive-offloading technologies (generative AI tools such as ChatGPT), which were primarily associated with reduced authorship and epistemic dependence; and institutional learning infrastructures (LMS, university platforms), which shaped context but were less frequently mentioned as sources of ambivalence (cf. Voinea et al., 2024; Skulmowski, 2023).

The longitudinal design enabled examination of stable and evolving aspects of technology dependence, alienation, and reflective awareness throughout the semester. This analytic strategy is consistent with recent applied IPA studies that demonstrate how meaning-making unfolds over time through idiographic and interpretative analyses of lived experience (Starr and Smith, 2022). MAXQDA 2022 supported systematic coding and data management, enhancing transparency and rigor. Trustworthiness was ensured through methodological triangulation, member checking with participants, collaborative coding, and reflexive practices. These strategies collectively enhanced the credibility and analytical depth of the findings (Lincoln and Guba, 1985). The second coder was a PhD candidate in psychology trained in IPA who independently coded 25% of the transcripts; inter-coder agreement on thematic categories was 84%, with discrepancies resolved through discussion. This contribution did not meet authorship criteria and is acknowledged in the Acknowledgments section.

## Results

Based on the longitudinal IPA design in this study, six key themes emerged that shed light on the subjective, cognitive, and emotional aspects of university students’ relationships with digital technologies. These themes illustrate that digital technologies are not merely tools but are experienced as reshaping students’ attention, memory, perception of time, and identity. The following six themes are: (1) From Habitual Engagement to Cognitive Embodiment: The Internalization of Digital Technology; (2) The Fragmentation of Cognitive Focus: The Collapse of Attention Regimes; (3) Loss of Control and Addiction-Like Engagement: Perceived Dependence on Digital Tools; (4) Disconnection from Meaning: Digital Acceleration and Its Impact on Memory and Meaning-Making; (5) Erosion of Subjective Integrity: Identity Alienation in Digital Learning; and (6) Reclaiming the Pharmakon: Moments of Remedial Engagement. Each theme is detailed below, grounded in participant narratives, with brief interpretive links to the study’s conceptual frame where analytically necessary. Table 2 presents the longitudinal trajectories of all twelve participants across the three interview waves. The thematic analysis below draws on these trajectories, with quotations selected to illustrate both dominant patterns and counter-tendencies. Consistent with the functional distinction between attention-capturing, cognitive-offloading, and institutional technologies outlined in the Method, Themes 1–2 draw predominantly on attention-capturing technologies such as smartphones and social media, Themes 3–5 foreground cognitive-offloading technologies such as generative AI tools, and institutional learning infrastructures surface more diffusely across the themes as a shaping context rather than as a discrete driver of dependency.

**Table 2:** Longitudinal trajectories across three interview waves (*n* = 12).

Participant	Week 1 (Baseline)	Week 8 (Midpoint)	Week 16 (Endpoint)	Trajectory type
P1	Intentional use; clear boundaries	Fragmented attention during exams	Partial recovery; renewed awareness	Recovery
P2	Structured reading plan	Compulsive checking; distraction	Continued struggle; insight	Decline with insight
P3	Conscious use; critical stance	Automatic AI use; “getting by”	Identity alienation	Linear decline
P4	Efficient use; moderate engagement	Overreliance on AI summaries	Disconnection from meaning	Decline
P5	Planned use	Multi-screen switching	Reduced retention	Decline
P6	Motivated; self-directed	ChatGPT dependence; superficial learning	“Someone else learned”	Linear decline
P7	Low use; rural context	Reaching for phone without reason	Recognition of habit; collaborative study group	Aware but stable
P8	High use; efficiency focus	Compulsive checking	Acceptance	Stable high use
P9	Handwritten notes; resistance	Return to AI under exam pressure	Cyclical relapse and adaptation	Cyclical
P10	Low use; rural context	Mental blocks; distraction	Disengagement	Decline
P11	High use; writing with AI	Loss of authorship	Identity erasure	Linear decline
P12	Moderate use	Physical tension; distraction	Exhaustion	Decline

### From habitual engagement to cognitive embodiment: the internalization of digital technology

This theme explores how students’ engagement with digital technologies has gradually evolved into an automatic, unconscious behavior, becoming deeply integrated into their cognitive structures. Initially adopted for everyday activities such as entertainment, social interaction, and staying informed, digital technologies have become entrenched in students’ routines, with their use evolving into repetitive, unquestioned habits alongside academic life. Over time, technology has ceased to be merely a tool; it has evolved into an automatic process embedded within students’ cognitive flow. In this context, technology shapes behavior through internalization, influencing attention management and even physical movements.

Most participants described this habituation process as a gradual transformation rather than an abrupt shift. P3 shared that, while in high school, she used social media only in her free time. However, she now “*opens it without even realizing what I’m doing or watching during class, not for any specific reason, but as if my brain is on autopilot*,” indicating that her use has become a reflexive behavior rather than intentional. Similarly, P8 remarked, “*If I do not have my phone, I feel something is missing. I open it without knowing why*,” highlighting the instinctive nature of his behavior. P5 shared, “*As soon as I sit down, my hand reaches for my pocket. I do not even know what I’m checking; I just pick up my phone and glance at whatever’s there*,” which further illustrates how this habit has become automatic, even at a physical level. P10 reflected on how internalization occurred quietly over time: “*My friends used it, so I started using it too. But I did not realize when it had become an addiction. I never thought I could get to this point*.” Some participants linked this change to a shift in their perception of time. P2 explained, “*When I use my phone, I lose track of time, unaware of how quickly it passes. I do not know when I started or what I did. I’m just wasting time*,” revealing how technology has altered their experience of temporality. P7 also observed, “*After being away for a while, I realized I was constantly reaching for my phone, wasting time on meaningless things*,” noting how they later recognized the automatic nature of these behaviors. These experiences suggest that digital technology has become more than just a tool. It has become an integral part of students’ cognitive and temporal infrastructures. Technology is no longer merely a medium for accessing information or for communication; it has transformed into a holistic mental process that fills emotional voids, manages time, and temporarily compensates for perceived emotional and existential gaps. Participants described this transformation as leading to notable cognitive consequences, such as reduced attention spans, increased distractibility, and weakened internal thought processes.

This internalization process can be read in dialogue with Stiegler’s concept of technology as a pharmakon, in which technology serves both as a cognitive enhancer and as a force that mechanizes cognitive functions (Stiegler, 2013b). As demonstrated by the participants’ experiences, technology assumes a dual role: it supports cognitive functions while simultaneously mechanizing them, reshaping temporal rhythms and attention economies by aligning them with external systems. Stiegler’s notion of tertiary retention helps explain this shift, illustrating how digital systems have, over time, become decisive in shaping individual memory and thinking patterns (Stiegler, 2013a). The shifts in participants’ perceptions of time and their behavioral automatism can be understood as consequences of this technical temporality. In Stieglerian terms, participants’ accounts can be read as indicating that algorithmic continuity may suppress internal temporal structures, aligning the rhythm of attention with external technical systems and, over time, weakening self-awareness, attention capacity, and desire-driven orientations.

### The fragmentation of cognitive focus: the collapse of attention regimes

This theme examines how students’ attention spans have fragmented over time, weakening their ability to concentrate on academic tasks and contributing to mental fatigue through intensive interaction with digital technologies. Participant narratives reveal that exposure to technological stimuli has led to shorter and more fragmented attention spans, hindering deep learning. Smartphones, social media, AI applications, and multitasking across multiple screens were experienced by participants as disrupting academic productivity and cognitive continuity. Participants described this fragmentation as not only reducing attention but also depleting motivation, decreasing learning efficiency, and fostering dependence on constant digital stimulation, thereby disrupting cognitive rhythm.

At the beginning of the semester, most participants reported no difficulty focusing on academic tasks, but concentration became increasingly difficult as time progressed. P2 described this process as follows: “*At first, I stuck to my reading plan. But then I started spending more time on social media. I check my phone five to ten times for every page I read and cannot focus on anything*.” Similarly, P6 said, “*I used to reflect while taking notes. Now, I just copy information from other sources. It’s harder to concentrate. Everything feels superficial now*.” P10 said he experienced mental blocks, especially as exams approached. “*My head feels full all the time, yet empty simultaneously. I cannot focus on anything. The computer is on, but my hands and mind are on my phone for no apparent reason. My brain is like a ship floating on the sea*.” Some participants also described the physical effects of this distraction. For example, P7 said, “*Even my shoulders tense up when I try to read something. I’m constantly stressed. It feels like I need to switch tasks while staring at the screen*.” P12 described this feeling as follows: “*It’s like there are always tabs open in my mind. Before I can finish one thought, another alert pops up. Sometimes, I even forget what I was thinking*.” P1 emphasized how distraction impacts productivity: “*I’ve noticed that when I get distracted, my productivity drops. It takes me three hours to do an hour’s worth of work. Yet, I still check social media. I do not know if I’m looking for something new or if it’s just a habit. I cannot break free from this trap*.” P5 reflected on this change in attention span: “*I used to make plans, but now I jump between screens. I want to learn everything quickly, but cannot retain the information*.” These narratives suggest that participants experienced a breakdown not only in attention but also in subjective rhythm, depth of thought, and the desire to learn. Over the semester, participants described their mental resilience as weakening and themselves as becoming entrenched in a cognitive pattern characterized by rapid responses. This cycle, compounded by constant technological stimuli, accelerates the collapse of attention regimes.

This experience aligns with Bradley’s (2015) reading of Stiegler, which frames the condition as ‘hyper-solicitation’, where digital systems continuously capture attention and prevent the stabilization of focus required for deep learning. It also resonates with Stiegler’s account of attention capture and the reorganization of desire within digital pharmacological systems (Stiegler, 2013b). The data suggest that the attention economy does not merely distract but also reorganizes attention and desire at the level of pre-reflective engagement, replacing deep attention with reactive engagement loops. From a Stieglerian perspective, these accounts indicate that the attention economy of digital capitalism fundamentally reshapes not only learning but also the nature of thinking itself.

### Loss of control and addiction-like engagement: perceived dependence on digital tools

This theme examines how students’ interactions with digital technologies, initially intended to simplify their academic tasks, can evolve into addiction-like patterns of engagement, resulting in a perceived loss of self-regulation. Over time, what began as a tool for convenience and productivity has gradually become an essential component of academic work, leading to increased reliance on such technologies. This escalating dependence has created a cycle in which the tools meant to assist in learning now contribute to mental exhaustion, feelings of alienation, and diminished productivity. In many cases, students reported that their academic practices, such as researching, writing, and problem-solving, had become intrinsically tied to these digital tools. This shift represented a change in how they worked and perceived themselves as learners, ultimately reducing their capacity for independent thought and cognitive effort.

Most participants reported that they initially used technology to facilitate their academic tasks but eventually became unable to function without these tools. For example, P6 explained how technological dependence became an integral part of their academic routine: “*ChatGPT is the first thing I open when doing my homework. I feel like I cannot write anything without it*.” This dependency led to internal conflict for others, as P2 highlighted the emotional struggle: “*Sometimes I get angry with myself. It bothers me that I cannot do things on my own, but there’s nothing I can do about it*.” Similarly, P11 expressed a deeper sense of alienation, stating, “*It’s like technology is learning, not me. I’m just following along. It feels like my mind has been taken out of the equation. It’s as if it’s doing the homework for me*.” As the dependence grew, participants also described the toll it took on both their mental and physical well-being. For instance, P12 conveyed how this reliance led to a sense of exhaustion: “*I get tired, but I cannot stop. It feels as though my brain has surrendered to technology. After a while, I cannot get back to my own rhythm*.” This loss of control over technology use was echoed by P3, who noted the shift toward automatic behavior: “*I used to use technology consciously. Now, my hand automatically reaches for AI every time. I like getting solutions quickly, but ultimately, I do not know how much I’ve learned. Honestly, I can say I’m just getting by*.” Despite these challenges, some participants made efforts to regain control. P9, for example, attempted to limit AI usage by taking notes by hand at the beginning of the semester, but eventually reverted to old habits during exam time, saying, “*I returned to the same method again because I did not have enough time*.” P7 shared a similar experience, stating, “*I try to work on my own, but when time runs short, I give up. After a while, you miss that convenience*.” These accounts reflect a profound shift not only in students’ academic practices but also in their sense of subjectivity. Digital tools were described as accelerating production but also as leading participants to transfer their intellectual autonomy, cognitive effort, and desire-driven orientations to external systems. This phenomenon leads to a paradigm shift in learning, in which active production is replaced by passive consumption.

These narratives suggest a perceived transfer of epistemic agency from students’ own cognitive labor to external technical systems, an interpretation consistent with Stiegler’s account of proletarianization (Stiegler, 2013a). The experiences captured in this theme illustrate how students’ ability to produce knowledge as active subjects diminishes, leaving them reliant on external algorithms. This highlights what Moore (2018) describes as systemic “pharmacological toxicity,” where technology short-circuits the long-term circuits of desire, reducing them to immediate drives. Following Heaney (2020), these addiction-like patterns are interpreted not as individual failures of will but as outcomes of a technical milieu that captures attention and desire through drive-based feedback loops. While it offers a seemingly attractive solution, it also functions as a pharmakon that weakens subjective production and displaces epistemic agency.

### Disconnection from meaning: digital acceleration and its impact on memory and meaning-making

This theme examines how participants experienced the intensive use of digital technologies as impairing their ability to retain meaningful information, affecting their memory, cognitive continuity, and the construction of personal meaning in learning. Although digital technologies provide quick access to information, their speed hinders deep comprehension and personal internalization. AI-driven platforms, short-form videos, and algorithmic content encourage students to form fleeting, superficial connections with information. Consequently, students often focus merely on “accessing” or “consuming” information, struggling with the deeper cognitive processes necessary for producing meaning and retaining information in the long term.

At the beginning of the semester, P6 found technology helpful for saving time; however, as the semester progressed, he struggled to maintain attention and retain information. He explained, “*I watch videos and do assignments, but when the same thing comes up again, I remember nothing and have to start over*,” highlighting his difficulty with memory retention. Similarly, P1 experienced fragmentation of focus, especially during exams: “*I have five tabs open at once. Information pours in from everywhere, but I do not fully grasp any of it. I glance at everything, think I understand, but I have to start over the next day*.” P8 added to this by pointing out the pedagogical consequences: “*The learning process has accelerated, but my comprehension has slowed down. I consume everything but internalize nothing*.” This sense of fleeting memory was echoed by P11, who noted, “*I gather information from everywhere, but it does not feel like mine. After an exam, my mind resets itself*.” P3 expressed a similar frustration with learning, saying, “*I can solve a coding problem, but afterward, I cannot remember the underlying logic. I apply the method but do not truly grasp it*.” P10 summarized this pattern well: “*I believe I’m learning through technology, yet I fail to internalize it. Everything remains external to me*.” Finally, P4 described the disconnect between information and understanding: “*While studying, I watch short videos on YouTube, but later, when we discuss the topics in class, nothing comes to mind*.” Initially, most participants thought that digital technology enhanced their efficiency. However, by mid-semester, the rapid pace of learning led to an increasingly superficial understanding. By the end of the semester, students reported heightened mental fatigue, distraction, and emotional disengagement in their learning, leading to a broader sense of alienation from the process of meaning-making.

These findings resonate with Stiegler’s account of tertiary retention and symbolic misery (Stiegler, 2013a). In this framework, participants’ descriptions can be interpreted as a growing reliance on externalized retrieval that prioritizes immediacy over internalization. This diminishes the role of secondary memory, which underpins personal experience and genuine meaning-making. As reflected in participant narratives, learning experiences no longer reside within the students’ subjective inner worlds; instead, they become externalized, fragmented, and ephemeral digital traces. This erosion of meaning was experienced as profoundly affecting both the retention of knowledge and the subjective value attributed to learning, corresponding to what Stiegler terms symbolic misery, where the exteriorization of knowledge into digital archives impoverishes interior life (Stiegler, 2013a). Over time, this results in diminished motivation, increased detachment from educational pursuits, and a reduced sense of intrinsic value of learning. In this light, technology operates as a pharmakon that simultaneously supports access to knowledge while eroding the subjective conditions necessary for durable memory and meaning-making (Stiegler, 2013a, 2013b).

### Erosion of subjective integrity: identity alienation in digital learning

This theme explores how students’ intense and continuous interactions with digital technologies lead to the disintegration of their self-recognition as learners, their sense of intellectual effort, and active engagement in learning processes. Initially perceived as tools that enhance productivity, facilitate quick access to information, and promote autonomy, digital technologies have, over time, led students to gradually distance themselves from their own creations, intellectual reflections, and subjective decision-making processes. Participants describe this phenomenon not only as an issue of efficiency but also as a form of identity disintegration, a weakening of the connection between the self and the act of creation, and a loss of cognitive ownership.

P2 shared, “*I used to get excited when working on projects and would dive into research. Now, the first thing I do is ask AI. I like the quick solution, but sometimes I’m not sure if it’s really mine*.” This highlights the growing blurring of the relationship between the student and their own production. Similarly, P11 reflected, “*I no longer write in my own words. It feels like someone else is learning, and I’m just watching. Sometimes, after an exam, I do not know what I’ve learned or who actually learned it*.” This comment points to the weakening connection between personal experience and genuine knowledge acquisition. In a similar vein, P4 stated, “*We live in a copy-paste world. It’s not about learning; it’s about appearing to have produced something. Adapting to technology has become easier than being myself*,” pointing out that adapting to digital systems has become a higher priority than maintaining one’s sense of self. P7 echoed this sentiment, stating, “*I used to think while taking notes. Now, AI summarizes for me. I read it, but the thinking process is no longer mine*,” indicating that cognitive engagement has been replaced by external processing. P9 further emphasized this loss of personal connection, saying, “*Everything is in my hands, but I am not. In this process, I feel like I’ve lost my own voice, my own perspective*,” emphasizing the identity erasure in the learning process. P3, similarly, stated, “*Technology was a power for me at first, but then it became something that guided me. Now, I do not even know how I learn*,” expressing the externalization of individual control over her learning. P6 summarized this gradual shift as follows: “*At first, I was very motivated. I made my own plan, took notes, and prepared questions. But after the middle of the semester, I started looking for shortcuts. Looking back, it feels like someone else learned in my place*.” These narratives collectively illustrate that learning is not just an instrumental activity but also a deeply existential relationship with the self. Over time, however, this connection with one’s own learning process begins to erode.

This process of perceived disintegration can be conceptualized through Stiegler’s notions of proletarianization and libidinal economy. As participants described algorithmic systems guiding their intellectual and creative activities, they experienced these systems as externalized not only capacities for knowledge production but also their desires and self-construction. According to Stiegler, when individuals lose the ability to identify with their cognitive labor, they cease to be subjects and instead become objects of technological systems (Stiegler, 2013a). The experiences captured in this theme indicate that participants experienced digital tools as leading them from production to consumption, and from thinking to mere application. Thus, while still involving the acquisition of knowledge, the learning process simultaneously signifies the dissolution of subjective integrity. In this light, digital technology does not merely serve as a functional learning tool but also acts as a pharmakon, restructuring the subject, reshaping their desires, and transforming their cognitive effort (Stiegler, 2013b). Although it offers convenience and speed, it was experienced as simultaneously diminishing students’ capacity to form and sustain their own identity.

### Reclaiming the Pharmakon: moments of remedial engagement

While the dominant trajectory across the semester was toward fragmentation, dependence, and alienation, several participants also described moments in which digital technologies functioned as a genuine remedy rather than a poison. These counter-tendencies are analytically crucial because they demonstrate the pharmacological ambivalence at the heart of Stiegler’s framework: the same technical supports that capture attention and desire can also, under certain conditions, sustain learning, care, and symbolic production.

P9, for example, described an attempt to reclaim slower, more deliberate study practices: “*I tried to take notes by hand for the first few weeks. It felt like my thoughts had more space. I could actually think before writing*.” Although she eventually returned to AI-assisted methods under exam pressure, this moment illustrates that the pharmakon is not deterministic: participants actively negotiate their relationship with technology.

P7, despite reporting compulsive checking, also described a collaborative study group that met weekly to discuss readings without devices: “*We leave our phones in our bags and just talk about the texts. It’s the only hour of the week when I feel like I’m actually learning for myself*.” This practice represents what Stiegler calls a reinvestment in symbolic production—a fragile but real counter-tendency to symbolic misery.

P2 similarly described a moment of what he called “*technological detox*” during a weekend trip: “*I left my phone at home. At first I felt anxious, but after a day I started noticing things again. I wrote in a notebook. It was like my brain woke up*.” This account suggests that the pharmacological balance can shift when the technical environment itself changes.

These counter-cases do not negate the dominant crisis narrative. Rather, they complicate it by showing that digital wellbeing is not simply a matter of abstaining from technology but of reconfiguring the conditions under which technology is used. As Stiegler (2013b) insists, the pharmakon is never resolved once and for all: it must be continually cared for through practices of attention, collective meaning-making, and pedagogical support. These moments of remedial engagement do not represent a resolution of the pharmacological crisis but rather its inherent openness: the same technical supports that capture attention can, under conditions of care, collective practice, and pedagogical support, sustain symbolic production and desire.

## Discussion and conclusion

In this study, the transformative effects of digital technologies on the subjectivation, attention, memory, and desire structures of higher education students were examined within Stiegler’s philosophical framework of the pharmakon. The findings suggest that digital technologies significantly influence students’ cognitive processes, attention management, and learning motivations, reflecting the pharmacological ambivalence emphasized by Stiegler (2010, 2013b).

The analysis indicates that digital technologies disrupt attention regimes, leading to a state of continuous stimulation and fragmented attention. This condition is defined in the literature as “hyperattention,” typically marked by rapid task-switching, preference for high levels of stimulation, and sustained engagement across multiple information streams (Bradley and Kennedy, 2019; Lee, 2019; Keller et al., 2020). While Skulmowski (2023) identifies this as mental fatigue, the present study interprets these findings, drawing on Bradley (2015), Stiegler’s account of libidinal economy (Stiegler, 2013b), and Moore (2018), as structural effects of a pharmacological environment that prioritizes drive-based reinforcement over desire and long-term projects. However, this study emphasizes that attentional fragmentation affects not only academic productivity but also weakens the processes of subjectivation, foregrounding a broader crisis of subjectivation that accompanies these seemingly mundane attentional shifts. With respect to usage patterns, high-intensity technology use was more frequently reported among participants from urban backgrounds; given the small, purposively selected sample and the descriptive rather than analytical status of this variable (see Table 1), this pattern should be read as an observation warranting dedicated investigation in future research rather than as evidence of a socio-spatial distribution of pharmacological vulnerability.

Participants described reduced permanence in memory and meaning-making due to digital environments, consistent with previous findings concerning superficiality of digital content and cognitive overload (Rozgonjuk et al., 2020; Lo Coco et al., 2020; Venn et al., 2020). However, this study revealed that superficiality is not merely a cognitive phenomenon but also a threat to individuals’ capacities for meaning-making and desire orientation, which relates to Stiegler’s concept of symbolic misery (2013a). Narratives of enduring study communities or shared interpretive work were comparatively rare across the dataset, which is consistent with a weakening of the symbolic and communal dimensions of learning that would otherwise support collective desire and transindividuation; however, as the counter-tendencies discussed below illustrate, this scarcity reflects a dominant pattern rather than a uniform absence. While symbolic misery has been primarily discussed in cultural and social contexts in earlier studies (Belgrano, 2024; Letiche et al., 2023), this research brings the concept into focus within individual learning experiences.

Importantly, the findings also revealed fragile but meaningful counter-tendencies to this dominant crisis narrative. Participants such as P7 and P2 described moments of reclaimed symbolic production—a phone-free study group, a weekend digital detox—that functioned as tentative reinvestments in care and collective meaning-making, while P9’s oscillation between handwritten note-taking and AI-assisted methods illustrates that the pharmakon is not a deterministic trajectory but a site of ongoing negotiation. These findings empirically substantiate Stiegler's (2013b) insistence that the pharmakon is never resolved once and for all, and they caution against reading digital technologies as uniformly toxic. Rather, they point to the conditions—collective practice, institutional support, deliberate slowing—under which the same technical supports can shift from capturing attention to sustaining it.

Furthermore, increased automatization and loss of control in students’ interactions with digital tools have been associated in the literature with the concept of proletarianization in Stiegler (2013a), which denotes alienation from one’s own production processes (Seo et al., 2021; Baker and Hawn, 2021). This study provides phenomenological evidence that proletarianization operates not only at cognitive and productivity levels but also at the level of identity integrity and desire integration. Thus, the study empirically supports Stiegler’s (2013a) perspective that technology profoundly transforms processes of subjectivation by penetrating individuals’ internal structures rather than serving as an external tool.

Finally, the findings of this research are consistent with previous studies indicating that digital technologies can trigger psychological issues among students, including depression, anxiety, loneliness, and burnout (Nan et al., 2024; De Oliveira Araújo et al., 2020; Twenge, 2020; Huang, 2020). However, unlike correlational studies that link psychological outcomes primarily to the duration of technology use, this study adopts a phenomenological approach that examines how these relationships are lived and interpreted in academic life.

In conclusion, this study proposes that digital technologies should be regarded as pharmacological systems transforming fundamental subjective structures such as attention, memory, desire, and identity in higher education, beyond their role as mere pedagogical tools (Stiegler, 2013b). At the same time, the counter-tendencies identified in this study indicate that this pharmacological effect is not fixed: under conditions of collective practice and sustained support, the same technologies can be reoriented toward care and symbolic production. Educational policies and digital pedagogical approaches must be assessed not only by criteria such as access, flexibility, and performance but also by their impact on students’ processes of subjectivation. Consequently, addressing these challenges requires more than digital abstinence; it necessitates reconstructing the “therapeutics” of attention in order to re-enable the symbolic and desire-driven dimensions of learning (Stiegler, 2010; Bradley, 2021).

### Limitations and future research

While this study provides an in-depth longitudinal, idiographic account of students’ experiences with educational technologies through an IPA lens, several limitations should be acknowledged. First, the sample comprised twelve participants. This cohort size is consistent with IPA’s commitment to depth and case-based interpretation; however, the findings should be understood as exploratory and context-specific, offering analytic and theoretical insights rather than statistically robust generalizations, and should be treated as rich empirical indicators rather than as a viable test of hypothesized relationships. Accordingly, the patterns identified here warrant examination in larger, more diverse samples, using complementary qualitative and/or mixed-methods designs to assess the prevalence, variation, and boundary conditions of these dynamics. Future research should also investigate how the pharmacological effects described in this study may differ across institutional arrangements, disciplinary cultures, and geographical contexts, thereby strengthening the transferability of the findings and refining the proposed conceptual implications for digitally mediated higher education. These implications are proposed as conceptually grounded directions rather than universal prescriptions.

## Data Availability

The raw data supporting the conclusions of this article will be made available by the authors, without undue reservation.
